# Triple-layer dissection of the lung adenocarcinoma transcriptome – regulation at the gene, transcript, and exon levels

**DOI:** 10.18632/oncotarget.4810

**Published:** 2015-09-02

**Authors:** Min-Kung Hsu, I-Ching Wu, Ching-Chia Cheng, Jen-Liang Su, Chang-Huain Hsieh, Yeong-Shin Lin, Feng-Chi Chen

**Affiliations:** ^1^ Department of Biological Science and Technology, National Chiao-Tung University, Hsinchu, Taiwan; ^2^ Institute of Population Health Sciences, National Health Research Institutes, Zhunan, Taiwan; ^3^ National Institute of Cancer Research, National Health Research Institutes, Zhunan, Taiwan; ^4^ Graduate Institute of Cancer Biology, China Medical University, Taichung, Taiwan; ^5^ Department of Biotechnology, Asia University, Taichung, Taiwan; ^6^ Center for Molecular Medicine, China Medical University Hospital, Taichung, Taiwan; ^7^ Cloud Computing and System Integration Division, National Center for High-Performance Computing, Taichung, Taiwan; ^8^ Department of Dentistry, China Medical University, Taichung, Taiwan

**Keywords:** lung adenocarcinoma, transcriptome analysis, alternative splicing, differential expression, transcript-specific regulation

## Abstract

Lung adenocarcinoma is one of the most deadly human diseases. However, the molecular mechanisms underlying this disease, particularly RNA splicing, have remained underexplored. Here, we report a triple-level (gene-, transcript-, and exon-level) analysis of lung adenocarcinoma transcriptomes from 77 paired tumor and normal tissues, as well as an analysis pipeline to overcome genetic variability for accurate differentiation between tumor and normal tissues. We report three major results. First, more than 5,000 differentially expressed transcripts/exonic regions occur repeatedly in lung adenocarcinoma patients. These transcripts/exonic regions are enriched in nicotine metabolism and ribosomal functions in addition to the pathways enriched for differentially expressed genes (cell cycle, extracellular matrix receptor interaction, and axon guidance). Second, classification models based on rationally selected transcripts or exonic regions can reach accuracies of 0.93 to 1.00 in differentiating tumor from normal tissues. Of the 28 selected exonic regions, 26 regions correspond to alternative exons located in such regulators as tumor suppressor (GDF10), signal receptor (LYVE1), vascular-specific regulator (RASIP1), ubiquitination mediator (RNF5), and transcriptional repressor (TRIM27). Third, classification systems based on 13 to 14 differentially expressed genes yield accuracies near 100%. Genes selected by both detection methods include *C16orf59*, *DAP3*, *ETV4*, *GABARAPL1*, *PPAR*, *RADIL*, *RSPO1*, *SERTM1*, *SRPK1*, *ST6GALNAC6*, and *TNXB*. Our findings imply a multilayered lung adenocarcinoma regulome in which transcript-/exon-level regulation may be dissociated from gene-level regulation. Our described method may be used to identify potentially important genes/transcripts/exonic regions for the tumorigenesis of lung adenocarcinoma and to construct accurate tumor vs. normal classification systems for this disease.

## INTRODUCTION

As the most deadly human cancer in the world, lung cancer was responsible for 1.59 million deaths in the year 2012 alone [1]. Lung adenocarcinoma is the most common subtype of non-small cell lung cancer [2, 3], which accounts for ∼85% of lung cancer cases. Gene dysregulation plays an important role in the development of lung adenocarcinoma [4, 5]. Multiple oncogenic pathways, including those pertaining to apoptosis and cell cycle control, are activated in lung adenocarcinoma tissues [6]. Accordingly, gene expression profiles have been suggested to be a good indicator of lung cancer prognosis [6–8]. Associations between gene dysregulation and tumorigenesis indicate that transcriptomic profiles could be suitable for distinguishing normal from tumor tissues [9].

A major goal of lung cancer studies is early clinical detection of the disease. Numerous molecular methods have been developed for this purpose. For instance, the serum concentrations of CEA and CYFRA 21-1 have been proposed as markers of lung cancer [10, 11], although they have not been successful in clinical trials [12]. Other approaches for diagnosing lung cancer include immunobiomarker tests [13–15] and the detection of DNA methylation of specific genes [16–18]. A few of these tests have been clinically investigated, but most of the proposed biomarkers for lung cancer, whether genomic, transcriptomic, epigenomic, or proteomic, have failed to yield satisfactory outcomes [12]. One important difficulty in biomarker selection regards the biological variation among tumor samples. Such variation has limited the sensitivity and specificity of the proposed methods [19–21]. Biomarkers and mathematical models to account for these variations have remained elusive.

Recently, transcriptomic approaches have been widely applied to the identification of biomarkers in lung cancer [22]. Most previous transcriptomic approaches have compared the gene expression profiles between tumor and normal tissues without considering the expressions of individual transcript isoforms. However, because most multiexon human genes are alternatively spliced [23], it is crucial to understand the contribution of transcript-specific regulation to the tumorigenesis of lung adenocarcinoma. The expression level of a gene is the summation of the expression levels of all of its transcript isoforms. Theoretically, the relative abundance of these transcript isoforms may differ substantially between tumor and normal tissues without affecting the overall gene expression level. Furthermore, during tumorigenesis, the expression level of a specific transcript may fluctuate more or less dramatically than that of the corresponding gene. Indeed, recent publications have highlighted the importance of transcript-specific regulations in tumorigenesis [24–29].

Aberrant splicing has been implicated in apoptosis, evasion from immune surveillance, cell proliferation, the Warburg effect, angiogenesis, metastasis, and response to anticancer drugs [30, 31]. The Cancer Genome Atlas (TCGA) Research Network revealed that somatic mutations could lead to important alterations in mRNA splicing in lung adenocarcinoma patients [20]. These findings suggest that mRNA splicing may represent a critical regulatory system in tumorigenesis worthy of further exploration.

Studies on the roles of splicing in tumorigenesis have mainly relied on exon array technology [32–37]. Unlike general microarray technology, exon arrays detect changes in exon- or transcript-level expression during cancer development [33, 38]. For example, four of the 5183 alternative exons examined by Misquitta-Ali *et al.* were differentially expressed between lung cancer and normal tissues [36]. Langer *et al*. used exon arrays with redesigned probes to identify 330 differentially spliced exons between non-small cell lung cancer and normal tissues [33]. The large difference between these two studies illustrates the limitation of exon array-based studies. These arrays rely on predesigned probes that can detect only a subset of exons/transcripts that were annotated at the time of probe design. In contrast, RNA sequencing (RNA-seq) does not rely on predefined probe sets and, therefore, is less biased in terms of transcript isoform detection. The development of RNA-seq technology has permitted the identification of numerous previously undiscovered transcript isoforms [39], many of which were undetectable or undistinguishable by exon arrays. Therefore, it is necessary to reexamine transcript-/exon-level regulation in lung adenocarcinoma by using RNA-seq data.

Some methods have been developed for transcript-specific expression analyses. The most widely used of these methods is the Cufflinks package [40], in which mappable RNA-seq reads are assigned to gene transcript isoforms based on a likelihood model. The tool calculates the number of fragments or reads per kilobase per million reads (FPKM or RPKM) for each transcript based on the “effective transcript length”. According to Cufflinks, the expression level of a gene is equal to the sum of the expression levels of all of its transcript isoforms. The Cuffdiff module of Cufflinks detects genes/transcripts that are differentially expressed between conditions (*e.g*., tumor vs. normal) according to a negative binomial distribution model [40, 41]. The DESeq/DEXseq package detects differentially expressed genes/exonic regions by applying a similar distribution model [42, 43]. Whereas Cuffdiff relies on effective length-corrected FPKM/RPKM values, DESeq/DEXseq detects differential expression based on “read counts” that are mapped to the genes/exonic regions of interest.

Cufflinks provides the expression levels of “complete transcripts” for detection of differentially expressed transcripts by Cuffdiff. In contrast, DEXseq evaluates the read count-based differential expressions of individual “exonic regions”. Therefore, these two tool sets may yield very different results. Some exon-level information may be omitted in transcript-focused analyses because 1) an exonic region can be shared by multiple transcript isoforms, and 2) variations in exonic expression can be leveled off in the calculation of transcript-level expression. By applying both tool sets (Cufflinks/Cuffdiff and DESeq/DEXseq) to the analysis of lung adenocarcinoma transcriptomes, we may be able to compare the performances of different approaches and to evaluate the contributions of three different regulatory levels (gene, transcript, and exon) to the tumorigenesis of this deadly disease.

In this study, we identify transcriptional changes at these three levels between tumor and normal tissues by analyzing RNA-seq data from 77 lung adenocarcinoma patients. This “base dataset” is fairly heterogeneous, comprising paired normal and lung adenocarcinoma tissues of different pathological stages from patients of both genders of various ages with or without smoking history. Thus, the transcriptomes of different individuals are expected to vary considerably. We demonstrate that the differential expressions of genes, transcripts, and exonic regions can be used to differentiate tumor from normal tissues with high accuracies (0.9–1.0), despite the heterogeneity in tumor samples. Our results support the importance of transcript-/exon-level regulation in the tumorigenesis of lung adenocarcinoma. They also support the use of individual transcripts/exons as potential drug targets and biomarkers for lung adenocarcinoma diagnosis. We suggest that further studies be made to explore the yet-unclear biomedical effects of, and molecular mechanisms underlying, these differential expression events.

## RESULTS

### Differences in expression at the transcript and exon levels are prevalent between lung adenocarcinoma and normal tissues

**Figure 1:**
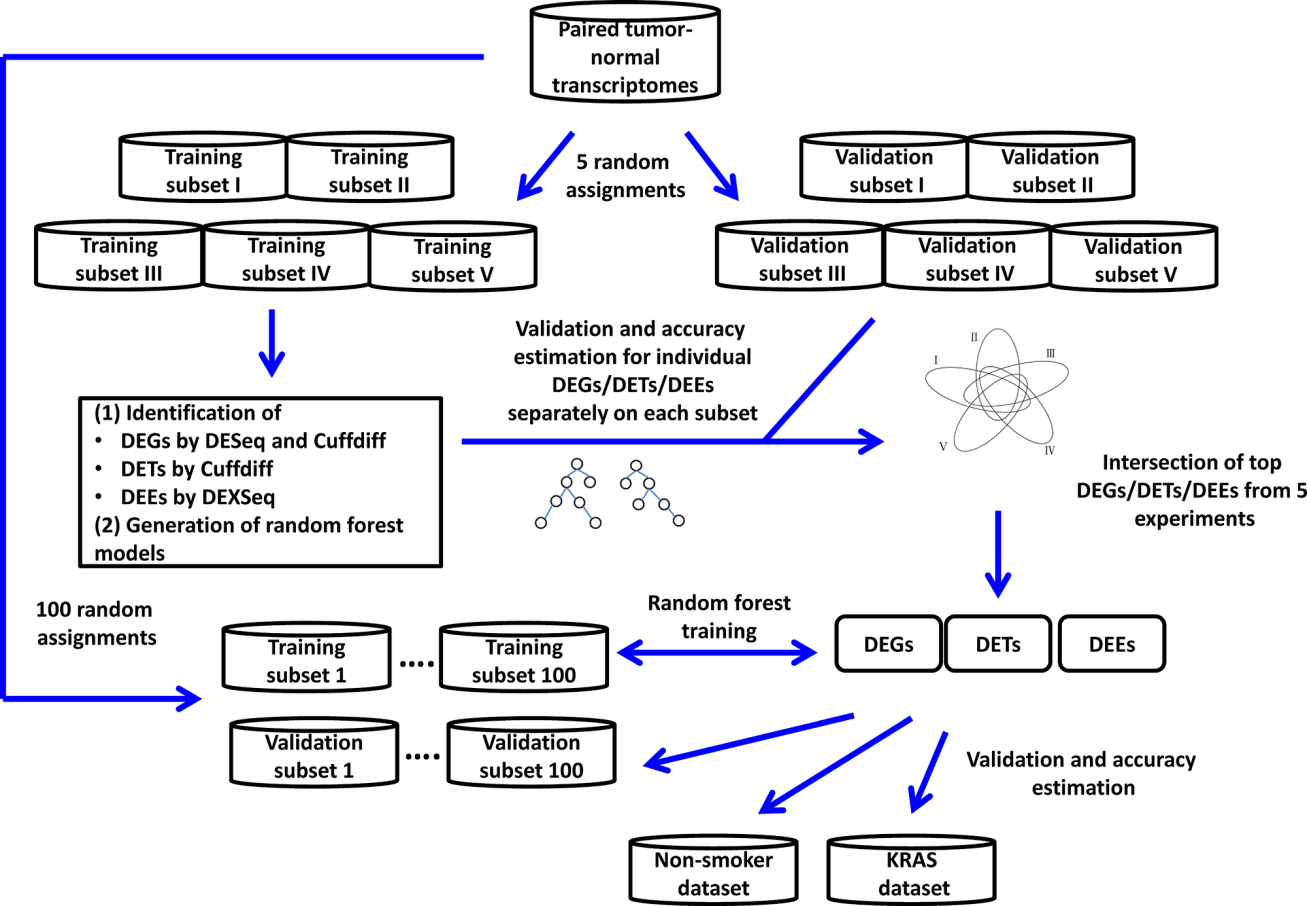
Analysis process of the study

Figure 1 presents the analysis procedure of this study. We randomly divided the transcriptomes of 77 patients (Supplementary Table S1) into training and validation subsets of approximately equal sizes. We compared the transcriptomes of paired tumor and normal tissues across 77 patients and used DEXseq and DESeq to identify differentially expressed exonic regions (DEEs) and genes (DEGs), respectively, in the training subset. For comparison, we used Cuffdiff to identify differentially expressed transcripts (DETs) and DEGs. Identified DEGs/DETs/DEEs were trained on the training subset to yield Random Forest models, and then tested on the validation subset for tumor vs. normal tissue classification accuracy. The random assignment of patients was repeated five times (referred to as “five-replicate experiments”) to account for between-individual variations in the transcriptome (Supplementary Table S2).

**Figure 2:**
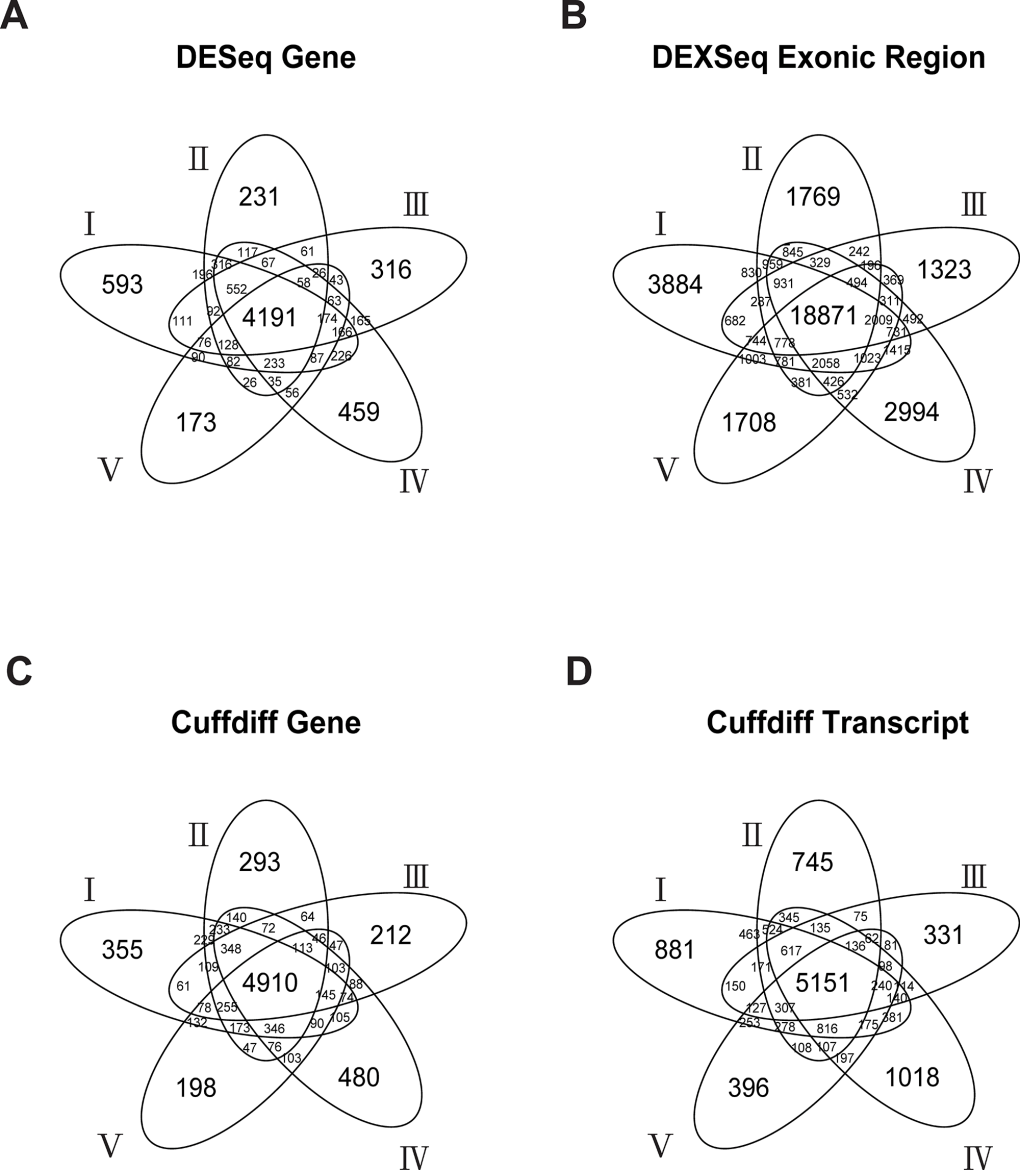
Numbers of DESeq-identified DEGs A DEXseq-identified DEEs B. Cuffdiff-identified DEGs C. and Cuffdiff-identified DETs D. in the five-replicate experiments (I–V)

When the Bonferroni-corrected cutoff *P* value was set as 0.05, we identified 5541 to 7313 DEGs and 28,789 to 36,986 DEEs by DESeq/DEXseq, and identified 6725 to 7643 DEGs and 7935 to 10,674 DETs by Cuffdiff in the five-replicate experiments (Figure 2). Intersections of the five replicates accounted for approximately 50% to 75% of the identified DEGs/DETs/DEEs. This between-patient heterogeneity in expression profile occurred at all three of the examined biological levels, suggesting that the sampling scheme could markedly influence the outcome of cancer-related transcriptome studies. Theoretically, DEGs/DETs/DEEs that occur recurrently in different sampling schemes should be important for lung adenocarcinoma tumorigenesis, and should be suitable features for constructing tumor vs. normal classification models. Thus, we used the DEGs/DETs/DEEs shared by the five replicates for subsequent analyses.

**Figure 3:**
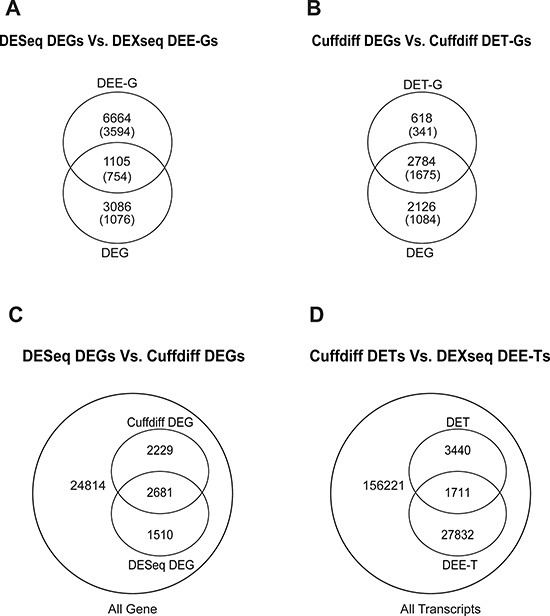
Intersection between DESeq-identified DEGs and DEXseq-identified DEE-Gs A. between Cuffdiff-identified DEGs and DET-Gs B. between DESeq-identified and Cuffdiff-identified DEGs C. and between Cuffdiff-identified DETs and DEXseq-identified DEE-Ts D Numbers in parentheses in (A) and (B) indicate the numbers of genes that are associated with cancer genes/characteristics according to MGSA.

The expression level of a gene is the sum of the expression levels of its exons/transcripts. Thus, the identified DETs/DEEs could reflect the differential expressions of the corresponding genes. If so, then the DETs/DEEs should be located mostly in DEGs, and transcript-/exon-level regulation should be functionally unimportant for lung adenocarcinoma tumorigenesis. To distinguish between gene-level and transcript-/exon-specific regulations, we mapped the selected DETs/DEEs to the corresponding genes (designated as DET-Gs and DEE-Gs, respectively). The 18,871 DEEs in the five-replicate intersection (Figure 1) could be mapped to 7769 DEE-Gs. Only 1105 (14.2%) of the DEE-Gs overlapped with the DESeq-identified DEGs (Figure 3A). Meanwhile, the 5151 DETs in Figure 1 corresponded to 3402 DET-Gs, of which 2784 (81.8%) overlapped with Cuffdiff-identified DEGs (Figure 3B).

We found that 182 of the 2784 DEGs were single-transcript genes, for which gene- and transcript-level regulations were virtually equivalent. The large difference between DET-Gs and DEE-Gs in overlapping with DEGs (81.8% vs. 14.2%) might have resulted partly from tool discrepancy and partly from the large variations in the estimation of exonic expression levels. Nonetheless, at least hundreds of transcript-/exon-specific regulatory events could be found in lung adenocarcinoma tissues. These observations suggest the existence of at least two layers of gene regulation in lung adenocarcinoma: (1) gene-centered regulation, in which genes are differentially expressed without changes in the relative abundance of transcript isoforms, and (2) transcript-/exon-specific regulation, in which transcript isoforms (or exonic regions) are differentially regulated without changing the overall gene expression level.

We also compared the results generated by different tool sets. At the gene level, Cuffdiff and DESeq significantly overlapped by 2681 genes (Figure 2, p < 2.2 × 10^−16^, hypergeometric test). These genes accounted for 54.6% and 64.0% of Cuffdiff- and DESeq-identified DEGs, respectively (Figure 3C). At the transcript level, DETs and DEE-Ts (the transcripts in which DEEs were located) significantly overlapped by 1, 711 transcripts (Figure 3D, p < 2.2 × 10^−16^, hypergeometric test), accounting for 33.2% of the Cuffdiff-identified DETs. The number of DEE-Ts was relatively large because one exonic region might be shared by multiple transcripts. The significant overlaps suggest that DESeq/DEXseq and Cuffdiff converged on differential expression events that are important for lung adenocarcinoma tumorigenesis.

Many differential expression events were identified by only one of the two tool sets. At the gene level, 2229 or 1510 DEGs were detected only by Cuffdiff or DESeq, respectively (Figure 3C). At the transcript level, 3440 Cuffdiff-identified DETs did not overlap with any DEXseq-identified DEEs, and 27,832 DEEs did not map to any DETs (Figure 3D). Therefore, the between-tool differences were substantial.

### Gene- and transcript-/exon-level regulations affect lung adenocarcinoma tumorigenesis through different mechanisms

To investigate the biological implications of the identified differential expression events, we conducted Model-based Gene Set Analyses (MGSAs) separately for the DESeq-identified DEGs and DEXseq-identified DEE-Gs in the five-replicate intersection (Figure 1), with reference to Gene Ontology (GO), Kyoto Encyclopedia of Genes and Genomes (KEGG) pathways, and Oncogenic Signatures (OSs) [44]. Results are shown in Supplementary Table S3.

The DEGs were enriched in five GO terms (multicellular organismal development, plasma membrane, cell cycle, cell proliferation, and receptor activity), and the DEE-Gs were enriched in six GO terms (nucleus, cytoplasm, transferase activity transferring phosphorus containing groups, biopolymer metabolic process, protein complex, and cell surface). Unexpectedly, none of the GO terms overlapped between DEE-Gs and DEGs. DEGs and DEE-Gs were enriched in 18 and 55 KEGG pathways, respectively, with six overlapping pathways (vascular smooth muscle contraction, pathways in cancer, axon guidance, cell cycle, extracellular matrix receptor interaction, and glutathione metabolism). DEGs and DEE-Gs were significantly associated with 27 and 39 OS genes/characteristics, respectively, with seven being shared by the two gene groups (*YAP* conserved signature, *MEK*, *CSR*, *VEGF-A*, *E2F1*, *EGFR*, and *RB*).

We conducted a similar analysis for Cuffdiff-identified DEGs and DET-Gs (Supplementary Table S3). Cuffdiff DEGs and DET-Gs were enriched in one and three GO terms as well as 18 and 10 KEGG pathways, respectively, and were significantly associated with 22 and 26 OS genes/characteristics, respectively. Cuffdiff DEGs and DET-Gs shared one GO term (cell cycle process), seven KEGG pathways (cell cycle, axon guidance, ABC transporters, purine metabolism, extracellular matrix receptor interaction, arginine and proline metabolism, and complement and coagulation cascades), and 17 OS genes/characteristics (Supplementary Table S3).

Collectively, the above observations suggest that DEE-Gs and DESeq-identified DEGs generally serve different biological functions. Although the biological functions of DET-Gs and Cuffdiff-identified DEGs overlapped considerably, the two gene groups actually diverged from each other by numerous biological features. Thus, we conclude that gene-level and transcript-/exon-level regulations can affect the tumorigenesis of lung adenocarcinoma through different molecular mechanisms.

### Ensembles of differentially expressed genes, transcripts, and exonic regions can differentiate tumor vs. normal tissues with high accuracy

Next, we prioritized the DEGs/DETs/DEEs for construction of tumor vs. normal classification models. We submitted individual DEGs, DETs, and DEGs identified in each of the five-replicate experiments to the Random Forest-based training process on the training data subsets. Then, we evaluated the accuracy of the resulting classification models on the validation data subset (Figure 1; Methods), and we ranked the DEGs/DETs/DEEs according to the accuracy of classification.

**Figure 4:**
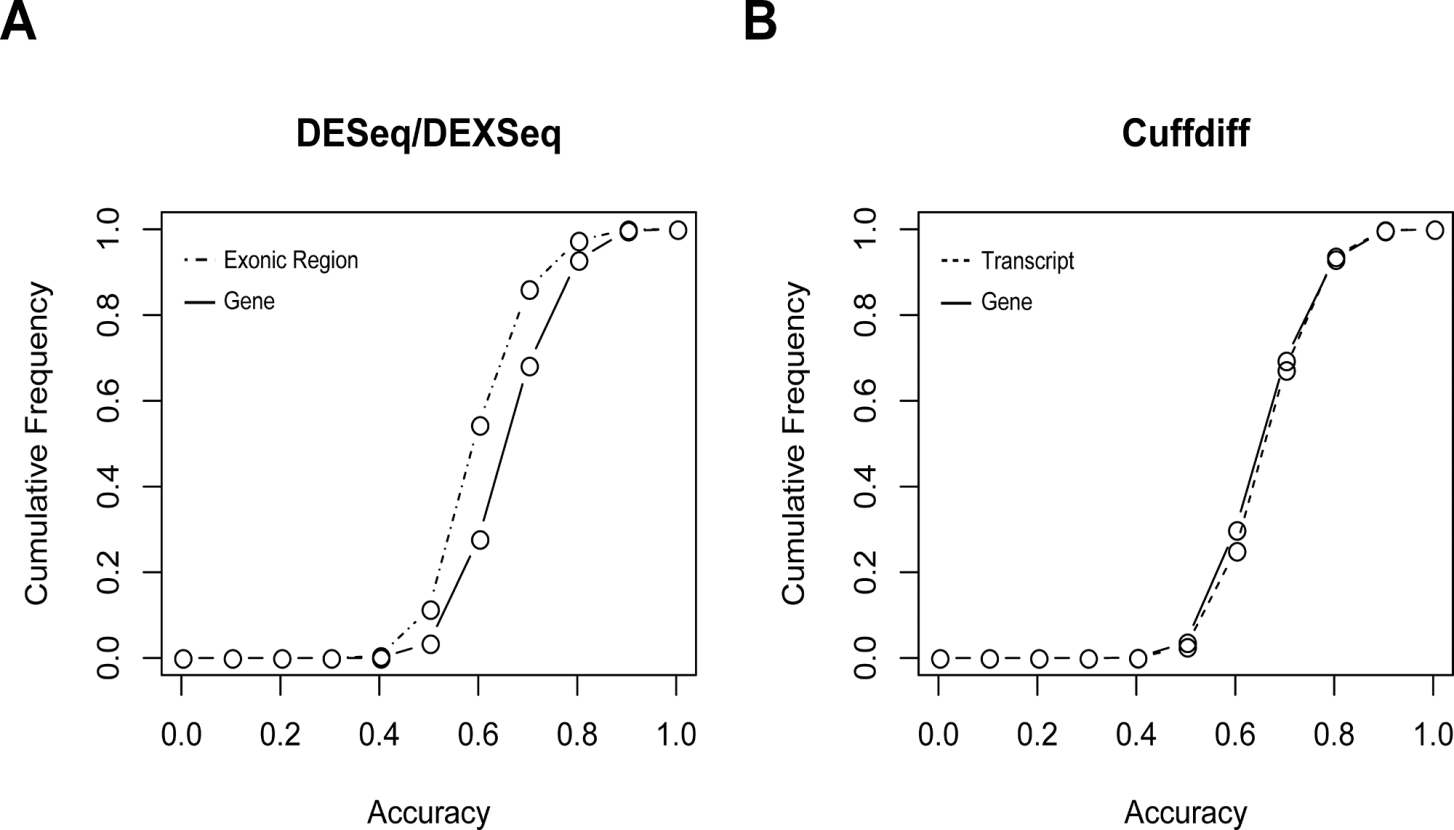
Cumulative frequencies of tumor vs. normal classification accuracy based on A. individual DESeq-identified DEGs or DEXseq-identified DEEs, and B. Cuffdiff-identified DEGs or DEGs This figure only shows the results derived from replicate I in the five-replicate experiments. Results of replicates II–V are given in Supplementary Figure S1.

Individually, DESeq-identified DEGs performed better than DEEs in classifying tumor vs. normal tissues (Figure 4A, Supplementary Figure S1), whereas the Cuffdiff-identified DEGs and DETs had very similar performances to each other (Figure 4B, Supplementary Figure S1). These similar performances may be attributable to the large overlap between the two sets of differential expression events (Figure 3B). Most (90%) of the single DEGs/DETs/DEEs yielded accuracies of less than 0.8, with single DEEs yielding the lowest accuracies (Figure 4). Thus, the level of exonic expression was generally more volatile than that of gene/transcript expression. In most cases, single DEGs/DETs/DEEs did not yield satisfactory tumor vs. normal classification results. Combinations of multiple differential expression events appear to be required.

The numbers of DEGs/DETs/DEEs analyzed were so large (>4, 000 genes/transcripts and > 18,000 exonic regions; Figure 2) that subsequent validation/discovery experiments would be difficult. In addition, some of these DEGs/DETs/DEEs might represent biological noise or sample-specific events. To obtain smaller but more biologically relevant sets of DEGs/DETs/DEEs, we applied more stringent selection criteria. Specifically, we reduced the cutoff *P* values for DEG/DET/DEE identification from 0.05 to 0.01 or 0.001, and retained only those that were ranked at the top 10%, 5%, or 1% of classification accuracy based on individual DEGs/DETs/DEEs. The three *P*-value cutoffs and three accuracy ranking cutoffs constituted nine different combinations of selection criteria (Supplementary Table S4). A “feature set” of DEGs/DETs/DEEs was derived under each combination of selection criteria. The combinatorial effect of each feature set on tumor vs. normal classification was evaluated in random assignment experiments.

We then conducted 100 random assignment or replicate experiments (in addition to the five-replicate experiments mentioned above) on the 77 patients. The 100 replicates were not gender-, smoking status-, or tumor stage-biased. For each replicate, the selected feature sets were trained on the training data subset, and the derived classification models were evaluated for accuracy on the validation data subset. One classification model could be obtained for each feature set in one replicate; therefore, each feature set could yield 100 classification models. Collectively, 100 models of one differential expression event (DEG, DET, or DEE) constituted a classification system. The models could “vote” to determine whether a specific sample should be classified as a “tumor” or as “normal” (Methods).

**Figure 5:**
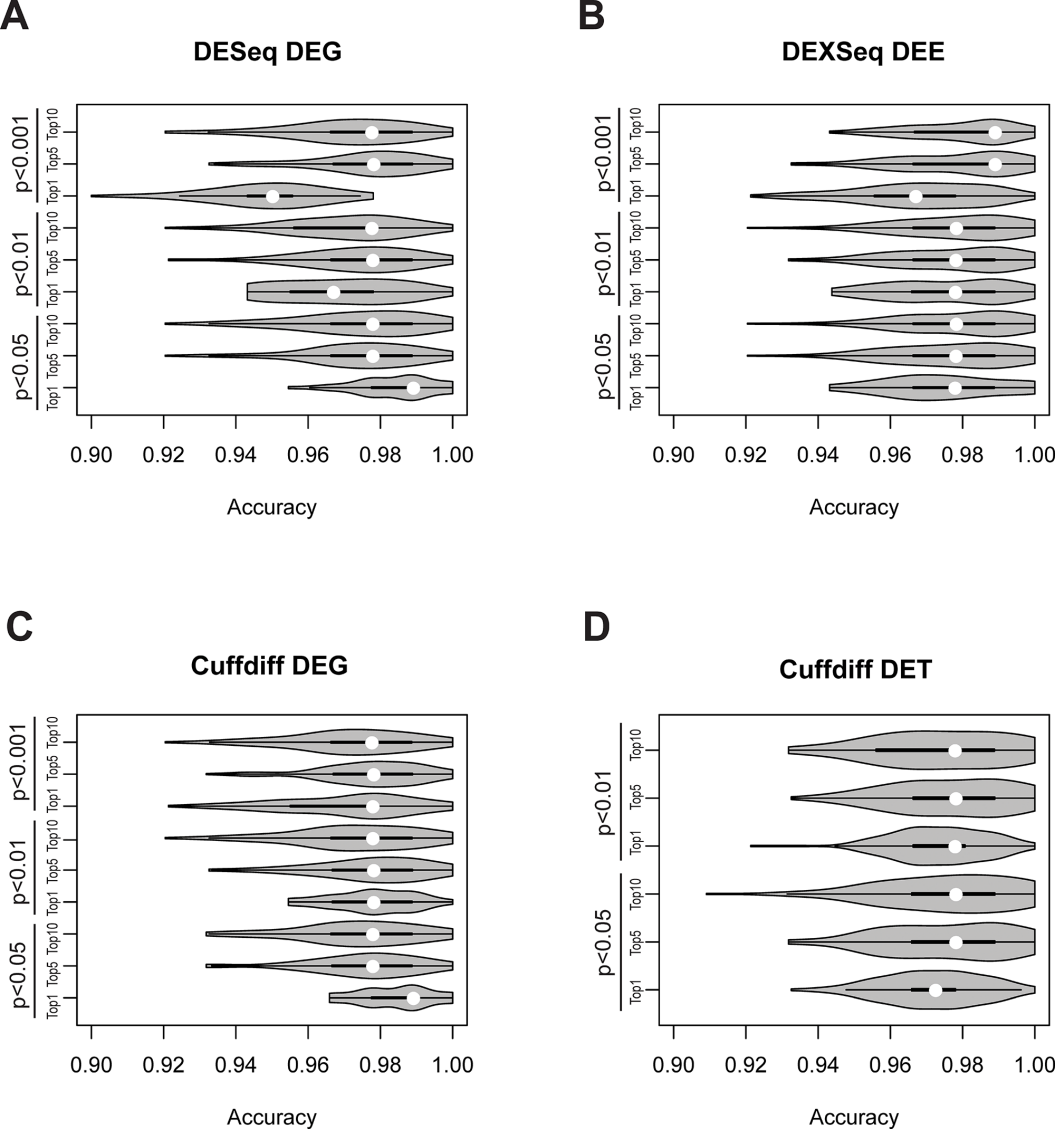
Violin-plot accuracy distribution of tumor vs. normal classification in 100-replicate experiments according to A. DEG-identified DEGs, B. DEXseq-identified DEEs, C. Cuffdiff-identified DEGs, and D. Cuffdiff-identified DETs White dots denote median classification accuracies.

Median accuracies of the DEG-, DET-, and DEE-based classification models were all higher than 0.95 across the nine selection criteria (Figure 5, Supplementary Table S4) and were substantially higher than accuracies based on individual DEGs/DETs/DEEs (Figure 4). For the Cuffdiff analysis, the *P* < 0.001 cutoff was so stringent that no DETs could be found. Therefore, only DETs obtained with cutoffs of *P* < 0.05 and *P* < 0.01 were analyzed. One of the DESeq-identified DEG feature sets yielded significantly lower accuracies than the other sets (Figure 5A). The lower accuracies probably resulted from information loss because this feature set (cutoff of *P* < 0.001, top 1%) included only two member genes (Supplementary Table S4). The highest classification accuracies were not necessarily derived from the most stringently selected feature sets. For the DESeq- and Cuffdiff-identified DEGs, the best-performing feature sets were derived from the *P* < 0.05 (top 1%) criterion, whereas the same selection criterion yielded the worst results for DETs. For DEEs, the highest accuracy was obtained under the criterion of *P* < 0.001 (top 5%).

Three conclusions can be drawn from these observations. First, combinations of DEGs/DETs/DEEs perform substantially better than individuals do in classifying tumor vs. normal tissues (Figures 4 and 5), especially for DEEs. Second, such combinations should include a sufficient number of DEGs/DETs/DEEs for accurate classification, but the accuracy may decrease if the number exceeds a certain threshold. Third, rationally selected DEGs/DETs/DEEs may reflect biological differences between tumor and normal tissues, at least in the base dataset.

The best-performing DEGs (cutoff of *P* < 0.05, top 1%) are listed in Table 1. Under this criterion, DESeq and Cuffdiff identified 13 and 14 DEGs, respectively, 11 of which were shared between the two feature sets. This high between-tool consistency implies that the regulation of these 11 genes is important for the tumorigenesis of lung adenocarcinoma. Moreover, some of these genes (*e.g*., *ETV4*, *RSPO1*, and *TNXB*) have been implicated in other tumors. Other molecular functions of the listed genes include roles as mediators, modifiers, activators, effectors, and inhibitors (Supplementary Table S5).

**Table 1:** DESeq- and Cuffdiff-identified DEGs selected for the tumor vs. normal classification systems

ENSEMBL ID	Identified by	Gene name	Associated cancer gene or pathway
ENSG00000144891	Cuffdiff	*AGTR1*	*EZH2* [53], *ESR1* [54]
ENSG00000132680	Cuffdiff	*KIAA0907*	*KRAS* [55], *TBK1* [55]
ENSG00000169241	Cuffdiff	*SLC50A1*	
ENSG00000254244	DESeq	*PAICSP4*	
ENSG00000135604	DESeq	*STX11*	*KRAS* [55], *STK33* [56], *IL2* [57]
ENSG00000162062	Both	*C16orf59*	*EDD* [53], *ALK* [58]
ENSG00000132676	Both	*DAP3*	cAMP [59]
ENSG00000175832	Both	*ETV4*	*EZH2* [53], *SUZ12* [53], *KRAS* [55], *IL15* [57], *CCND1* [60], *WNT* [61]
ENSG00000139112	Both	*GABARAPL1*	
ENSG00000128059	Both	*PPAT*	*NFE2L2* [62], *PIGF* [63]
ENSG00000157927	Both	*RADIL*	
ENSG00000169218	Both	*RSPO1*	*WNT* [61]
ENSG00000180440	Both	*SERTM1*	
ENSG00000096063	Both	*SRPK1*	cAMP [59], *EIF4GI* [64]
ENSG00000160408	Both	*ST6GALNAC6*	*WNT* [61], *AKT* [65]
ENSG00000168477	Both	*TNXB*	*KRAS* [55], *E2F1* [66]

### DEG-based classification systems outperform DET- and DEE-based systems on independent validation datasets

As the gene regulation of lung adenocarcinoma was heterogeneous between patients, we investigated whether classification systems derived from the base dataset could perform well when applied to other lung adenocarcinoma transcriptome datasets. We evaluated the accuracies of the DEG-, DET-, and DEE-based classification systems on two independent datasets: the “nonsmoker dataset” GSE37764 and the “*KRAS* dataset” GSE34914 (see Supplementary Table S1 for details). GSE37764 is composed of RNA-seq data of paired tumor-normal tissue samples from six nonsmoker female lung adenocarcinoma patients, with each sample being sequenced twice (24 transcriptomes in total). GSE34914 includes RNA-seq data of tumor samples (no normal samples) from 16 lung adenocarcinoma patients with or without *KRAS* mutations (one transcriptome per patient).

Using both datasets, we evaluated the accuracies of the 100-model classification systems based on the rationally selected DEGs/DETs/DEEs. A sample in the independent dataset was predicted to be tumor tissue if more than 50 of the 100 models in a system classified it as “tumor”. Otherwise, this sample was considered to be “normal”. Because the tumor-normal distinction is a binary variable, we used a continuous variable, system performance index (SPI), to evaluate the overall consistency of the four classification systems (DESeq DEG-based, Cuffdiff DEG-based, DET-based, and DEE-based systems). SPI was defined as the average proportion of models that correctly assigned a sample to “tumor” across all samples in a test dataset (Methods). An SPI value of 1.0 indicates that all 100 models correctly classify a tumor sample as “tumor”. An SPI value of ∼0.5 indicates that the tumor vs. normal classification is no better than random chance.

SPI values for the nonsmoker and *KRAS* datasets were 1.000 and 0.996 for the DESeq DEG-based classification system, 1.000 and 0.991 for the Cuffdiff DEG-based system, 0.995 and 0.948 for the DET-based system, and 0.998 and 0.919 for the DEE-based system, respectively (Table 2). For the nonsmoker dataset, the DEG-, DET-, and DEE-based systems all had SPI values of ∼1.000 (all of the tumor and normal tissues were correctly classified by all four systems). For the *KRAS* dataset, the two DEG-based systems had slightly higher SPI values (0.991–0.996) than the DET- or DEE-based system (0.919–0.948). These results imply that gene expression as a whole was more consistently regulated across tumor samples than either transcript isoform or exonic region expression. However, the biological significance of transcript-/exon-specific regulation in the pathogenesis of lung adenocarcinoma is not negligible, given the high accuracies of the DET- and DEE-based classification systems.

**Table 2:** SPI values of different classification systems on the nonsmoker and *KRAS* datasets

Feature set (#DEGs/DETs/DEEs)	Non-smoker (24 samples)	*KRAS* (16 samples)
DESeq DEGs (13)	1.000	0.996
Cuffdiff DEGs (14)	1.000	0.991
Cuffdiff DETs (50)	0.995	0.948*
DEXseq DEEs (397)	0.998	0.919*
DEXseq DEEs – Naïve FPKM (28)	0.930	1.000

* In the *KRAS* dataset, the DET- and DEE-based systems each made an error by assigning a tumor sample to the “normal” group, even though the SPI values were fairly high. All other predictions in this table reached 100% accuracy.

One tumor sample (SRR396813) in the *KRAS* dataset was erroneously predicted to be normal by both DET- and DEE-based systems (Supplementary Table S6). This result may be because this particular sample was an outlier in terms of its transcript-level expression profile (Supplementary Figure S2).

To investigate whether our method could be applied to other cancer types, we tested the method on prostate adenocarcinoma transcriptomes from The Cancer Gene Atlas website. This dataset included 104 tumor and normal tissue samples. We used 40 paired tumor-normal tissues (80 samples) to construct classification systems, as described above. The other 24 samples were used for validation. The resulting SPI values were comparable to those obtained from the lung adenocarcinoma analysis (Supplementary Tables S7 and S8). However, unlike in the lung adenocarcinoma analysis, the DEE-based system yielded the highest average SPI value (0.974; Supplementary Table S9) among the four classification systems. The DEE-based system correctly classified all 24 samples, whereas the other three systems misclassified one to three samples (Supplementary Table S8). These observations suggest that our method can be applied to other cancer types, although the overall consistency of tumor vs. normal classification and the relative performances of individual systems may differ between cancer types.

### The ETV4 gene is associated with cancer cell stemness in lung adenocarcinoma

**Figure 6:**
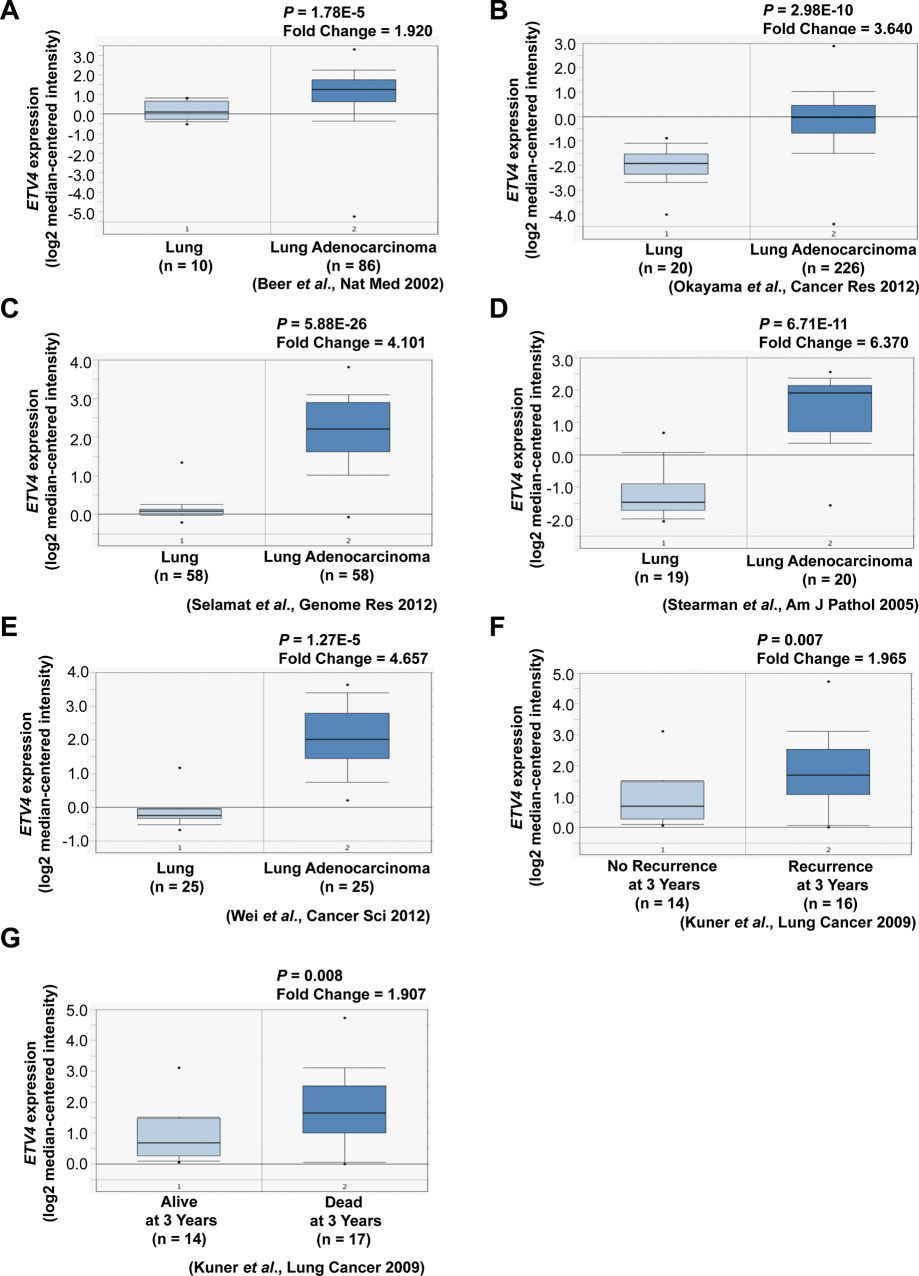
Association of *ETV4* expression with tumor, recurrence, and clinical outcomes of lung cancer patients **A–E.** *ETV4* mRNA expression in lung adenocarcinoma compared to normal lung tissues (Oncomine datasets: Beer, Okayama, Selamat, Stearman, and Wei Lung). **F.** *ETV4* mRNA expression as a function of lung cancer recurrence (Oncomine datasets: Kuner Lung). **G.** *ETV4* expression as a function of lung cancer patient survival (Oncomine datasets: Kuner Lung).

We examined whether our method could be used to identify genes important for the tumorigenesis of lung adenocarcinoma. Using the Ets variant 4 (*ETV4*) gene (Table 1) for functional analyses, we examined whether this gene is upregulated in lung adenocarcinoma by using an *in silico* approach based on the Oncomine database (https://www.oncomine.org/resource/login.html) [45]. We observed significant upregulation of *ETV4* in lung adenocarcinoma compared to normal lung tissues (Figure 6A–6E). Furthermore, *ETV4* upregulation was significantly associated with recurrence (Figure 6F) and poor survival (Figure 6G).

**Figure 7:**
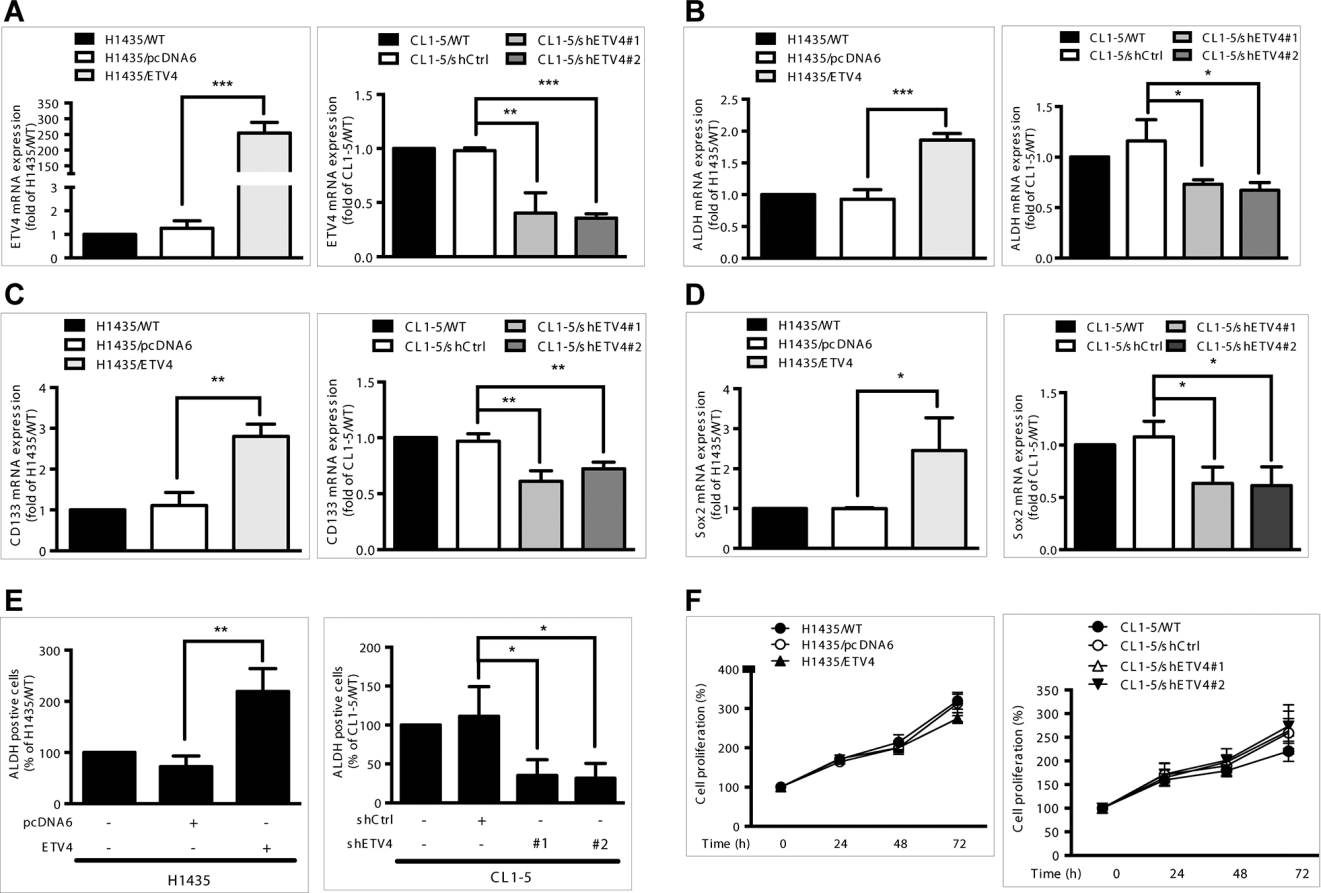
Effects of *ETV4* expression on lung cancer cell stemness and proliferation **A–D.** mRNA expression levels of *ETV4* (A), *ALDH* (B), *CD133* (C), and *Sox2* (D). **E.** *ALDH* activity as measured by flow cytometry. **F.** Percentage of cell proliferation as measured by the MTT assay in *ETV4*-overexpressed H1435 (left panel) and *ETV4*-knockdown CL1–5 cells (right panel). Results are shown as the mean ± standard deviation based on triplicate experiments. *P < 0.05, **P < 0.01, and ***P < 0.001 (Student's *t* test).

Next, we analyzed whether *ETV4* is correlated with cancer cell stemness or proliferation. After overexpressing *ETV4* using the pcDNA6 vector system or knocking down expression using *ETV4*-specific small interfering RNAs, we measured the mRNA expression levels of three stemness markers, *ALDH*, *CD133*, and *Sox2* (Figure 7A). *ETV4* overexpression or knockdown significantly increased or decreased, respectively, the expression levels of all three markers (Figure 7B–7D, left and right panels). Moreover, *ALDH* activity, a hallmark of cancer stem cells, was significantly increased or decreased in cells with overexpression or knockdown of *ETV4*, respectively (Figure 7F). Table 3 reports the correlations between the expressions of *ETV4* and the three biomarkers. Changes in *ETV4* expression did not significantly affect the proliferation of lung adenocarcinoma cells (Figure 7E). Taken together, these findings support the idea that *ETV4* could increase the stemness of lung cancer cells.

**Table 3:** Associations between expression levels of stem cell markers and *ETV4* in lung cancer datasets

Stem cell marker	Dataset	†*r*	‡*P*
ALDH1A1	Bhattacharjee (*n* = 203)	0.3904	< 0.0001
	Bild (*n* = 111)	0.2164	0.0225
	Broet (*n* = 72)	0.3614	0.0018
CD133	Broet (*n* = 72)	0.2689	0.0224
SOX2	Beer (*n* = 96)	0.2996	0.0051
	Bhattacharjee (*n* = 203)	0.1844	0.0117
	Ding (*n* = 75)	0.3304	0.0038
	Lee (*n* = 138)	0.3201	0.0001
	Stearman (*n* = 39)	0.5059	0.0219

† *r*, Pearson's correlation coefficient,

‡ *P* value for two-tailed Student's *t* test of individual dataset (Oncomine database [45]).

*n*, total sample size, including cell lines and normal and cancer tissues. Correlation was only assessed in the cancer sample.

## DISCUSSION

By comparing the transcriptomes of paired tumor and normal tissues, we identified hundreds to thousands of DEGs, DETs, and DEEs from tissues derived from 77 lung adenocarcinoma patients. In most cases, the identified DEEs did not coincide with the DEGs (Figure 3A), and hundreds of DETs did not correspond to DEGs. These observations imply that there is substantial exon-/transcript-specific regulation in lung adenocarcinoma. However, we cannot exclude the possibility that some of the identified DETs/DEEs represent background variations or biologically irrelevant events. Therefore, stringent criteria for the identification of DETs/DEEs are necessary.

With the differentially expressed events identified in the initial five-replicate experiments (Figure 2), we constructed classification systems (each comprising 100 Random Forest models) to differentiate tumor from normal tissues in lung adenocarcinoma patients. The DEG-, DET-, and DEE-based systems all yielded average accuracies not lower than 0.95 when tested on the validation data subsets (Figure 5, Supplementary Table S4). When applied to the nonsmoker dataset, all four systems reached 100% prediction accuracy, with slightly different consistency levels (SPI: 0.995–1.000, Table 2). When applied to the *KRAS* dataset, the DET- and DEE-based systems misclassified one tumor sample, but the two DEG-based systems classified all samples correctly. The SPI values of the DET- and DEE-based systems were lower than those of DEG-based systems for this validation dataset.

The high classification accuracies of all four systems for the nonsmoker dataset were not surprising, as the nonsmoker validation dataset was entirely derived from female nonsmoking Korean patients (Supplementary Table S1). Similar samples were included in the base dataset. The observation that DEGs could better overcome between-dataset variations implies that the regulatory flexibility of splicing and the variations in estimating transcript/exonic expression are larger than those of the overall gene expressions. Estimates of transcript- or exon-level expression may be reliable only for highly expressed genes [46]. Thus, removing transcripts of genes with lower expression might help to improve the consistency of DET-/DEE-based classification.

Large variations in transcript/exonic expression levels might have led to overfitting in the construction of classification models in the lung adenocarcinoma analysis. This overfitting may explain why the DET- and DEE-based systems made an error in the *KRAS* dataset. Furthermore, many more DETs and DEEs were selected for the classification systems compared to DEGs. This disparity could have aggravated the overfitting problem.

We further clarified how the classification error occurred by examining the expression profiles of SRR396813. Expression levels of DEGs in this specific tumor sample were closer to the medians of all samples than in the case of DETs, for which the expression levels were mostly at the high extremes (Supplementary Figure S2). This example illustrates that although the expression levels of individual transcripts may vary considerably, the expression levels of genes as a whole are relatively stable across patients. Nevertheless, we identified large numbers of DEG-independent DETs/DEEs in lung adenocarcinoma (Figure 3), which could be used to improve the characterization of the tumor tissues (Figure 5, Table 2). Studying the biological roles of these DETs/DEEs might advance our understanding of lung adenocarcinoma pathogenesis and aid researchers in identifying specific transcripts/exonic regions for the development of more precise therapeutics.

In the above analyses, the expression level of a DEXseq-identified DEE was represented by the expression level of the corresponding transcript, which, in turn, was estimated by using the effective length-correction algorithm implemented in Cufflinks [40]. This algorithm could assign reads to the supposedly “correct” transcripts, thus avoiding errors in estimating transcript expression levels. Cufflinks considers a transcript as a unit of expression. Thus, estimates of transcript expression can level off the variations in exonic expressions, which may be biologically meaningful.

We were interested in whether the uncorrected exonic expression levels could be helpful in classifying tumor vs. normal tissues. We substituted the FPKM values calculated by Cufflinks with the FPKM values that had not been corrected for effective length (naïve FPKMs) (Methods). Using the naïve FPKMs as inputs to DEXseq analyses in the five-replicate experiments, we identified 28 DEEs for the construction of another tumor vs. normal classification system. Of the 28 DEEs, 26 DEEs corresponded to alternatively spliced exons, whereas only two DEEs were constitutively spliced exons. Moreover, 18 of the 28 DEEs (64%) were found in only one of the transcript isoforms of the corresponding genes (Supplementary Table S7).

Next, we validated the 28-DEE–based classification system by applying it to the nonsmoker and *KRAS* datasets, revealing SPI values of 0.930 and 1.000, respectively (Table 2). This system correctly classified all of the tumor and normal samples in the *KRAS* dataset (Supplementary Table S6), in contrast to the 50-DET- and 397-DEE–based systems that erroneously assigned one tumor sample to “normal”. These 28 DEEs did not fall within any of the 13 DESeq- or 14 Cuffdiff-identified DEGs in Table 1. These observations indicate that the naïve FPKMs for DEEs could provide useful information for the tumor vs. normal classification.

One limitation of this study is that the base dataset included only patients of one single ethnicity (Korean). The uniform genetic background might limit the applicability of the classification models. This limitation is probably one reason why, in addition to the outlying expression profile of the specific sample, the DET- and DEE-based systems made an erroneous prediction in the *KRAS* dataset. However, both DEG-based systems correctly classified all of the samples. This result seems to suggest that DEG-based classification systems are more robust than DET-/DEE-based systems against genetic and biological variations in differentiating tumor vs. normal tissues.

Although the DEE-based system outperformed the DET-/DEG-based systems for the TCGA prostate adenocarcinoma dataset, relatively few DEEs/DETs/DEGs were identified in this dataset (only 2–9 events; Supplementary Table S8). The disparity in the numbers of identified DEEs/DETs/DEGs might have resulted from the difference in sample size between the two analyses. The number of tumor/normal samples for classification system construction was ∼50% smaller in the analysis of prostate vs. lung adenocarcinoma (80 samples vs. 154 samples). The high accuracy of the DEE-based system in the prostate adenocarcinoma analysis highlights the advantage of exploring exon-level transcriptional regulation. When limited numbers of differential expression events are available, DEEs could be useful for identifying tumor vs. normal differences with better accuracy than DETs or DEGs.

Overall, our analyses demonstrate that, in addition to genes, transcripts and exons can also serve to differentiate between tumor and normal tissues reliably. Furthermore, exonic expression levels might convey important regulatory information that is not observable in gene- or transcript-level expression. This triple-layered view of the cancer transcriptome can help to identify previously unexplored regulatory events that may be important for tumorigenesis, thereby facilitating future diagnostic/therapeutic developments.

## MATERIALS AND METHODS

### Data source

RNA-seq datasets of lung adenocarcinoma were downloaded from the Gene Expression Omnibus database under series numbers GSE40419 [47], GSE34914 [48], and GSE37764 [49] (Supplementary Table S1). The base dataset, GSE40419, was used to identify DEGs, DETs and DEEs between normal and tumor tissues. These differentially expressed events were submitted to the Random Forest module of the R package for construction of classification models to distinguish normal from tumor tissues based on the training data subsets (Figure 1). The base dataset contained RNA-seq data derived from adjacent normal and tumor tissues from 77 Korean patients, and RNA-seq data from tumor tissues of 10 patients. Only the transcriptomes of the 77 paired tissues were used for model training. Non-paired tissues were included for model validation only.

GSE34914 (the *KRAS* dataset) included 16 lung adenocarcinoma tumor samples, including eight samples that contained *KRAS* mutations and seven samples that did not. GSE37764 (the nonsmoker dataset) included paired tumor and normal tissues from six Korean female nonsmokers, with each sample being sequenced twice. The *KRAS* and nonsmoker datasets were used as independent validation datasets to evaluate the performances of the DEG/DET/DEE-based classification systems.

### RNA-seq data processing

Raw data retrieved from the Gene Expression Omnibus (in SRA format) were converted to the fastq format by using fastq-dump. RNA-seq reads were mapped to the human reference genome (GRCh37; Ensemble Version 70) by using TopHat2 with default parameters [50]. Expression levels (FPKM values) of genes and transcripts were generated by Cufflinks [40]. The expression level of an exonic region was calculated by summing the FPKM values of transcripts containing the exonic regions of interest.

To ensure data quality, we excluded two types of genes and the corresponding transcripts: 1) genes with multiple FPKM values (12 genes), and 2) genes for which some of the annotated transcripts were “absent”, rather than being assigned zero FPKM values, according to Cufflinks results (776 genes). For the five-replicate experiments (Figure 1), one additional filter was applied: the FPKM of a gene/transcript must be a non-zero value in the tumor and normal tissues of at least 39 patients. This filter was used to ensure the success of the Random Forest training process. If more than 38 (∼ half of the patients) zero FPKM values were assigned, to one single training data subset, then Random Forest training would be infeasible. After these exclusions, 31,234 genes and 150,132 transcripts were included in the subsequent analyses of 100 random assignments.

### Detection of regions with differential expression

Cufflinks can be used to estimate transcript/gene expression levels, but not the statistical significance of differential expression. Therefore, differential expression between tumor and normal tissues was evaluated by using DESeq (for genes) and DEXseq (for exonic regions) [42, 43]. We calculated the read counts of the genes/exonic regions of interest with the HTSeq python package, using the TopHat2 mapping results. The DESeq/DEXseq algorithm approximates a negative binomial distribution model based on the number of “counts” of mappable RNA-seq reads in multiple samples, and tests the statistical significance of the difference in read count between two conditions (in this study, tumor vs. normal tissues). To reduce variation in the read-count estimates, we discarded exonic regions that were shorter than 100 bp. For comparison, we used Cuffdiff to identify DETs and DEGs [41]. Cuffdiff employs a negative binominal distribution model based on Cufflinks-derived FPKM values to detect the differential expressions of genes/transcripts. Bonferroni-corrected *P* values of 0.05, 0.01, and 0.001 were used as cutoffs to identify DEGs, DETs, and DEEs.

### Calculation of naïve FPKM values and functional analysis of differential expression events

We calculated the naïve FPKM values from the RNA-seq reads that mapped to the DEEs detected by DEXseq, using the formula ${\text{FPKM}}_{i}=\frac{n_{i}}{n_{T}\times l_{i}}$, where *FPKM_i_* is the naïve FPKM of exonic region *i*, *n_i_* is the number of reads mapped to exonic region *i*, *n_T_* is the total number of mapped reads (in millions), and *l_i_* is the length of exonic region *i* (in kbp).

The R package “mgsa” was used to analyze the functional effects of identified differential expression events [44]. MGSA is a Bayesian modeling approach for gene set enrichment analysis with reference to GO, KEGG pathways, and OSs.

### Construction of tumor vs. normal classification systems

The R package “randomForest” [51] was used for the construction of tumor vs. normal classification models. Using this algorithm, we randomly selected candidate DEGs/DETs/DEEs to construct multiple decision trees with controlled variances. The trees then “voted” to determine whether a sample was a tumor or a normal tissue. Classification models were constructed from the training data subsets and tested for accuracy on the validation data subsets generated in the random assignments of the base dataset. Each random assignment could yield a Random Forest-trained classification model based on the input feature set of DEGs, DETs, or DEEs. A classification system was the combination of all 100 models based on the same type of differentially expressed events, which were derived from 100 random assignments of the base dataset.

### Calculation of SPI

The SPI value reflects the average consistency of a system in classifying tumor vs. normal samples. The confidence (*s_i_*) of sample *i* assigned to “tumor” was defined as $s_{i}=\frac{n_{\text{it}}}{n}$, where *n_it_* is the number of models that classify test sample *i* to tumor, and *n* is the total number of models (in this study, 100). If *s_i_* was larger than 0.5, then the sample was assigned to “tumor”; otherwise, the sample was assigned to “normal”. Precision index *r_i_* was defined as:

$$r_{i}=\{\begin{array}{c} {\text{s}}_{i}\text{if is a tumor sample;}\\ 1-{\text{s}}_{i}\text{if the sample is normal.} \end{array}$$

Finally, SPI was defined as $\text{SPI}=\frac{\sum_{i=1}^{N}r_{i}}{N}$, where *N* is the number of samples.

### Cell lines for experimental validation

Human cell lines used in this study included the lung adenocarcinoma cell line H1435 (American Type Culture Collection [ATCC] CRL-5870), the embryonic kidney cell line HEK-293T (ATCC CRL-3216), and the lung adenocarcinoma cell line CL1-5 (provided by Dr. Cheng-Wen Wu [52]). Cells were cultured in Dulbecco's Modified Eagle Medium/Nutrient Mixture F-12 (DMEM/F12; for H1435 and CL1-5 cells) or high-glucose DMEM media (HEK-293T cells) at 37°C in a humidified atmosphere containing 5% CO_2_. Media were supplemented with 10% fetal bovine serum (FBS), 2 mM L-glutamine, and 1% penicillin/streptomycin.

### Plasmid constructs and small hairpin RNA (shRNA) clones

Full-length human *ETV4* (NM_001986) was amplified from the cDNA of CL1-5 cells by polymerase chain reaction (PCR), using forward primer (F) gggaattcGG ATGGAGCGGA GGATGAA and reverse primer (R) gggcggccGC TGGGGGCTAG TAAGAGT, where gggaattc and gggcggcc were sequences including the restriction enzyme cutting site. The gene was cloned into the *EcoR*I and *Not*I sites of pcDNA6 (Invitrogen). *ETV4* shRNA clones (TRCN0000013933 and TRCN0000013936) and the luciferase shRNA control clone (TRCN0000072244) were purchased from the National RNAi Core Facility at Academia Sinica, Taipei, Taiwan. All plasmids were confirmed by DNA sequencing.

### Transfection and lentiviral infection

For overexpression of *ETV4*, H1435 lung cancer cells were transfected with *ETV4*-overexpressing plasmid or control vector (pcDNA6) for 48 h by using the Fugene Reagent (Roche). For lentiviral production, the envelope plasmid (pMD2.G), packaging plasmid (pCMV-deltaR8.91), and target gene (*ETV4* shRNA clones and luciferase shRNA control clone) were transfected into HEK-293 cells by using Polyethylenimine Reagent (Sigma) for 24 h. The media was changed to Complete Medium, which included 10% FBS, 2 mM L-glutamine, and 1% penicillin/streptomycin. After 48 h, the supernatant was collected and filtered by a 0.2-μm syringe filter (Pall Life Sciences). For lentiviral infection, CL1-5 cells were infected with 2 ml of lentiviral supernatant and 2 μl of polybrene (hexadimethrine bromide, Sigma) for 48 h. These transient transfectants were further assayed according to the purpose of each experiment.

### RNA isolation and quantitative reverse transcription PCR (qRT-PCR)

Total RNA was isolated by using Trizol (Invitrogen) and used as a template for reverse transcription into cDNA by using the M-MLV Reverse Transcription Kit (Invitrogen). To quantify mRNA expression, qRT-PCR was performed with a LightCycler 480 (Roche) for the following genes (primer pairs): *ETV4* (F: gcagtttgtt cctgatttcc a, R: actctggggc tccttcttg), *ALDH* (F: ccaaagacat tgataaagcc ataa, R: cacgccatag caattcacc), *CD133* (F: ggaaactaag aagtatggga gaaca, R: cgatgccact ttctcactga t), *SOX2* (F: atgggttcggtggtcaagt and reverse primer: actctggggctccttcttg), and *GAPDH* (F: agccacatcg ctcagacac, R: gcccaatacg accaaatcc). Relative gene expression was calculated by 2^−ΔCT^ (ΔCT = CT of target gene – CT of *GAPDH* gene; CT is the cycle threshold).

### Cell proliferation assay

Cell proliferation was assessed by the MTT (3-(4, 5-dimethylthiazol-2-yl) -2, 5-diphenyltetrazolium bromide) assay. Cells (4 × 10^3^ cells) were seeded in 96-well tissue culture plates and incubated for 6 h. MTT (5 mg/ml) was added for further 4 hours after the indicated time points. Supernatant was removed, and 100 μl DMSO were added to dissolve the crystal. The absorbance at 570 nm was measured with an enzyme-linked immunosorbent assay reader, with subtraction of background at 630 nm.

### ALDH activity assay

The *ALDH* activity of cells was measured by using the Aldefluor Kit (StemCell Technologies, Vancouver, BC, Canada), followed by flow cytometric analysis in the green fluorescence channel, FL1. The aldefluor-specific inhibitor, diethylaminobenzaldehyde, was added to the sample as a control to define the gates of *ALDH*+ region.

## SUPPLEMENTARY FIGURES AND TABLES




















