# Supplementary material for: Triple-layer dissection of the lung adenocarcinoma transcriptome – regulation at the gene, transcript, and exon levels
Source: Oncotarget. 2015 Sep 2;6(30):28755–73. doi: 10.18632/oncotarget.4810 (PMC4745690; doi:10.18632/oncotarget.4810)
Supplement: Supplementary file 1 [file oncotarget-06-28755-s001.pdf]

SUPPLEMENTARY FIGURES AND TABLES

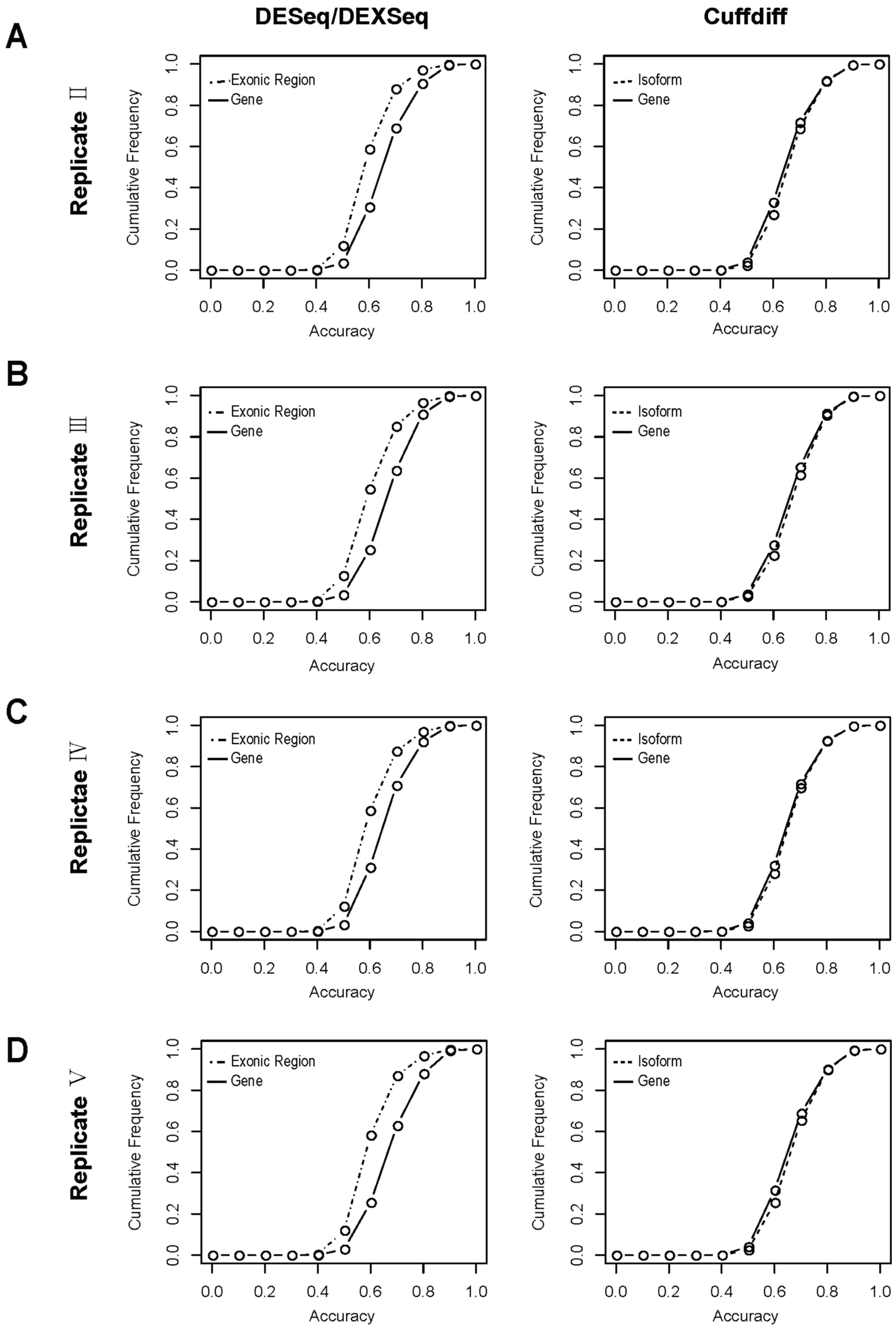

Supplementary Figure S1: Cumulative frequencies of tumor vs. normal classification accuracy based on individual DESeq-identified DEGs or DEXseq-identified DEEs (left column), and Cuffdiff-identified DEGs or DETs (right column) in replicates II–V A–D. of random assignments.

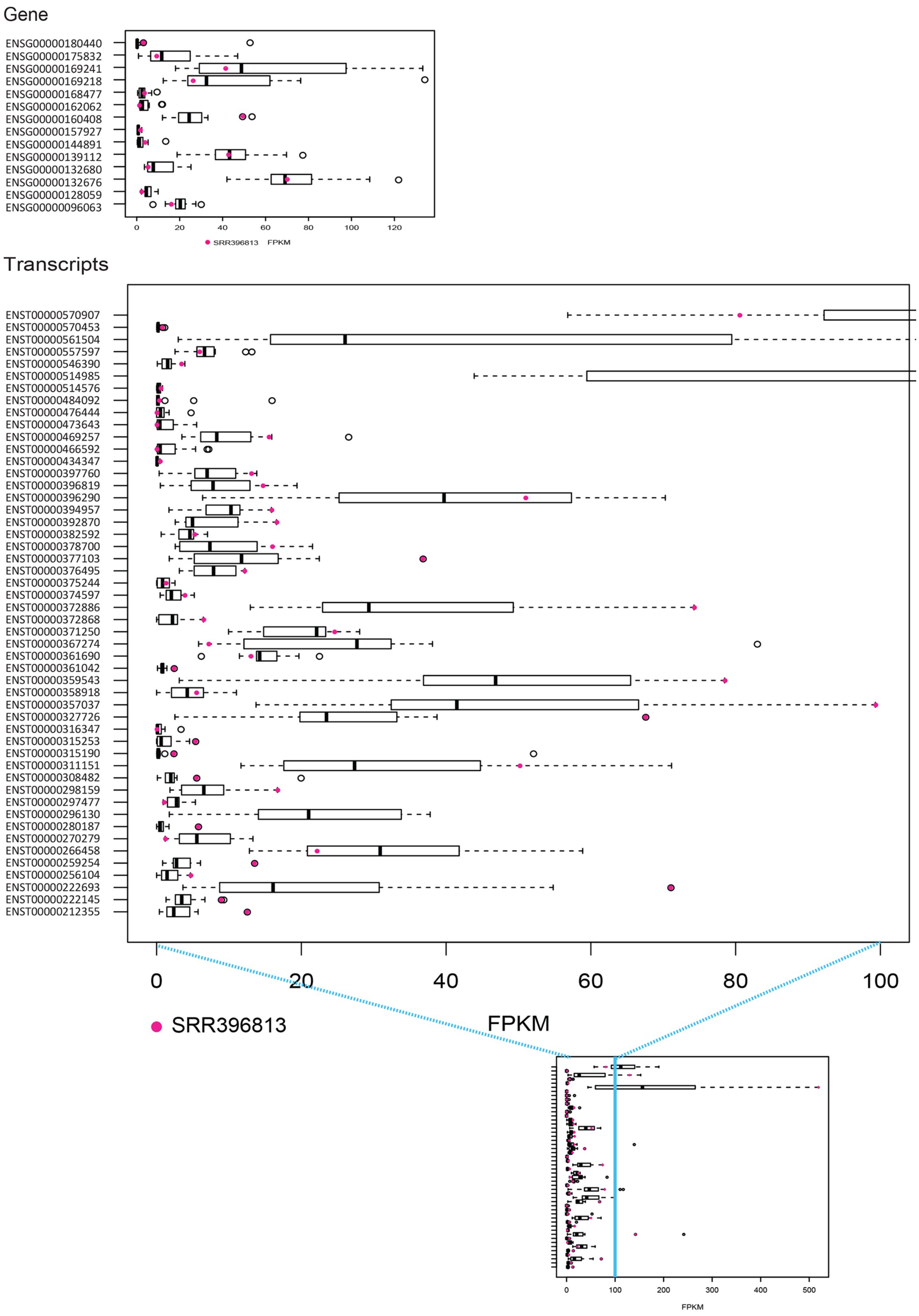

Supplementary Figure S2: Gene-level (upper panel) and transcript-level (lower panel) expression profiles of sample SRR396813 in the *KRAS* dataset. Boxes indicate the expression distributions of the 77 patients. Expression values of SRR396813 are shown as pink dots.

Supplementary Table S1: Datasets used in this study

Supplementary Table S2: Grouping of the five-replicate experiments

Supplementary Table S3: Biological functions of the identified DEGs/DETs/DEEs

Supplementary Table S4: The accuracies of 100-model classification systems in 9 selection criteria

Supplementary Table S5: Molecular functions of genes in Table 1 according to GeneCard annotations

Supplementary Table S6: Proportions of models (out of 100) that assigned test samples to the "tumor" group in two independent validation datasets

Supplementary Table S7: The 28 DEEs identified by using naïve FPKM values

Supplementary Table S8: Proportion of models (out of 100) that assigned the validation samples of prostate adenocarcinoma to the "tumor" group

Supplementary Table S9: SPIs of different classification systems on validation samples of prostate adenocarcinoma
